# Increase in the radioresistance of normal skin fibroblasts but not tumor cells by mechanical injury

**DOI:** 10.1038/cddis.2016.416

**Published:** 2017-02-02

**Authors:** Zelin Chen, Xin Wang, Taotao Jin, Yu Wang, Christopher S Hong, Li Tan, Tingyu Dai, Liao Wu, Zhengping Zhuang, Chunmeng Shi

**Affiliations:** 1 Institute of Combined Injury, State Key Laboratory of Trauma, Burns and Combined Injury, Chongqing Engineering Research Center for Nanomedicine, College of Preventive Medicine, Third Military Medical University, Chongqing 400038, China; 2 Surgical Neurology Branch, National Institute of Neurological Disorders and Stroke, National Institutes of Health, Bethesda, MD 20892, USA

## Abstract

The timing of radiation after mechanical injury such as in the case of surgery is considered a clinical challenge because radiation is assumed to impair wound healing. However, the physiological responses and underlying mechanisms of this healing impairment are still unclear. Here, we show that mechanical injury occurring before ionizing radiation decreases radiation-induced cell damage and increases cell repair in normal fibroblasts but not tumor cells *in vitro* and *in vivo*. At the molecular level, mechanical injury interrupts focal adhesion complexes and cell–cell cadherin interactions, transducing mechanical signals into intracellular chemical signals via activation of the phosphatidylinositol 3-kinase (PI3K), Akt, and glycogen synthase kinase 3 beta (GSK-3*β*) pathways. We show that subsequent nuclear translocation of nuclear factor (erythroid-derived 2)-like 2 (Nrf2) and *β*-catenin strengthen the stemness, antioxidant capabilities, and DNA double-strand break repair abilities of fibroblasts, ultimately contributing to increased radioresistance. Our findings demonstrate that mechanical injury to normal fibroblasts enhances radioresistance and may therefore question conventional wisdom surrounding the timing of radiation after surgery.

The potential for harmful radiation exposure has increased dramatically with the widespread application of radioisotopes in medicine and radiotherapy for cancer patients and possible threats from nuclear accidents or radiological terrorist attacks. Under such potential exposure scenarios, radiation often occurs in combination with other injuries, especially wound trauma. Previous studies have demonstrated that radiation exposure combined with wound trauma results in a negative synergistic effect that is much more harmful than either insult would produce alone.^1, 2^ Ionizing irradiation (IR) has long been known to greatly delay skin wound healing, exhibiting impaired inflammatory responses and decreased vascularity and collagen production.^3, 4, 5^ However, few studies provide a comprehensive understanding of such healing-impaired wounds. To provide more evidence of the physiological mechanisms underlying the effects of radiation on wound healing, we focus on the physiological response to IR combined with mechanical injury to fibroblasts, the main repair cells involved in wound healing. Surprisingly, the results showed that the order of occurrence of radiation and mechanical injury affected the outcome of combined effects such that survivals of fibroblasts improved when mechanical injury occurred prior to IR exposure. However, tumor cells did not show this alleviative combined effect. Further *in vitro* and *in vivo* investigation indicated that mechanical injuries decrease IR-induced cell damage and accelerate cell recovery. Mechanical injuries interrupt focal adhesion complexes and cadherin–catenin complexes, which in turn increases the stemness, antioxidant ability, and DNA double-strand breaks (DSBs) repair by activating the phosphatidylinositol 3-kinase (PI3K)/Akt/glycogen synthase kinase 3 beta (GSK-3*β*)/ Nuclear factor (erythroid-derived 2)-like 2 (Nrf2) and *β*-catenin signaling pathways, finally contributing to increased radioresistance. Our findings might provide investigators or doctors with alternative measures for treating combined injuries involving irradiation and wounds.

## Results

### Mechanical injury increases survival of fibroblasts following IR exposure

**Figure 1:**
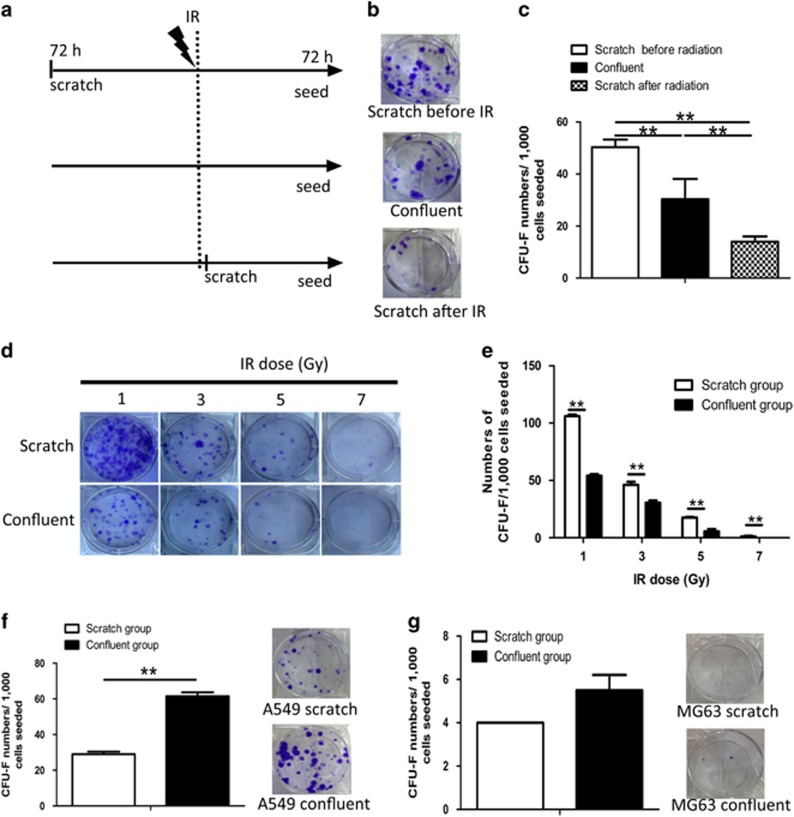
Mechanical injury increases survival of skin fibroblasts after IR. (**a**) Radiation and mechanical scratch scheme for skin fibroblasts grown to confluence over 7 days. (**b**) Representative pictures of surviving colonies of skin fibroblasts receiving only radiation, mechanical scratch before radiation, or mechanical scratch after radiation. (**c**) Quantification of skin fibroblast colonies in groups shown in **a**. (**d**) Representative images of surviving skin fibroblast colonies after exposure to radiation alone (1–7 Gy) or mechanical scratch before radiation. (**e**) Quantification of colonies shown in **d**. (**f**) Representative images and quantification of surviving colonies of A549 cells receiving radiation alone (5 Gy) or mechanical scratch before radiation (5 Gy). (**g**) Representative images and quantification of surviving colonies of MG63 cells receiving radiation alone (5 Gy) or mechanical scratch before radiation (5 Gy). **P<0.01. *P*-values were calculated using the one-way analysis of variance in **c** and the independent-samples *t*-test in **e**–**g**

To explore the combined effects of IR and wound trauma on fibroblasts, we used a mechanical scratch model to mimic wound injury, and cells were exposed to IR of 5 Gy before and after scratching. Cell survival was determined at 72 h after exposure to three different scenarios: mechanical injury followed by IR (post-wound IR), IR followed by mechanical injury (pre-wound IR), and IR alone without mechanical injury (Figure1a). Fibroblasts in the post-wound IR group formed the most colonies, while there were fewer colonies in the pre-wound IR group than in the IR alone group (Figures 1b and c). Fibroblast colonies in post-wound IR group were also more numerous than in the IR-alone group after exposure to different IR doses (1–7 Gy; Figures 1d and e). However, scratched A549 cells formed fewer colonies than confluent (unscratched) cells following 5 Gy IR (Figure 1f). Cell survival did not differ between scratched and unscratched confluent MG63 cells (Figure 1g). Furthermore, we compared the colony-forming ability of fibroblasts *in vivo* receiving wound trauma 3 days before IR to those receiving it immediately after IR. Granulation tissue-derived fibroblasts from the mice in the post-wound IR group formed more and larger colonies than those in the pre-wound IR group (Supplementary Figure S1a). These results indicated that mechanical injury occurring prior to IR increased radioresistance in human fibroblasts, while that occurring after IR had the opposite effect.^6^

### Mechanical injury decreases IR-induced DNA DSBs in human fibroblasts

**Figure 2:**
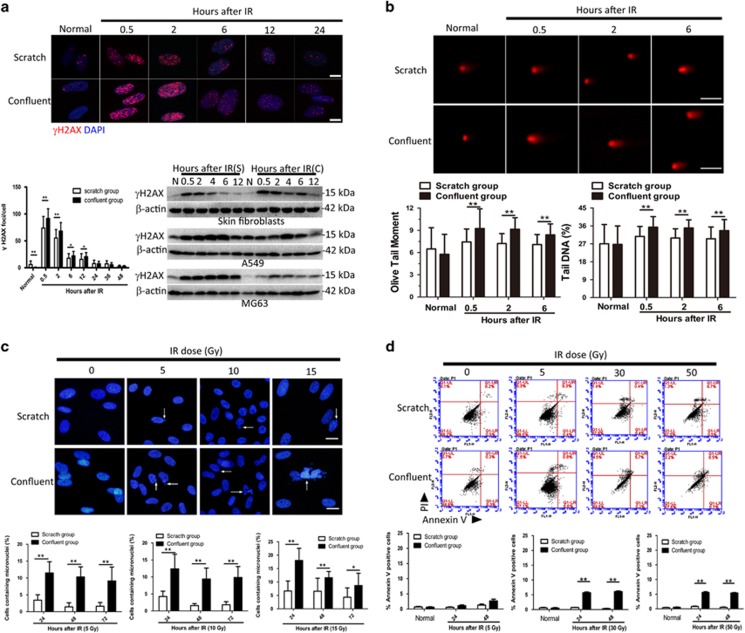
Mechanical injury decreases IR-induced cell damage and apoptosis in skin fibroblasts. (**a**) Nuclei of mechanically scratched and unscratched confluent skin fibroblasts were co-stained with *γH2AX* and 4′,6-diamidino-2-phenylindole (DAPI) at the indicated time points following radiation (5 Gy). Scale bars represent 20 *μ*m. Quantification of *γH2AX* foci is also presented. Western blot analysis of *γH2AX* expression in mechanically scratched or unscratched confluent cells (skin fibroblasts, A549 cells, and MG63 cells) following radiation (5 Gy) was performed. N, normal; S, scratched; C, confluent. (**b**) Distribution of comets in mechanically scratched or unscratched confluent skin fibroblasts at indicated time points following radiation (5 Gy). Scale bars represent 100 *μ*m. The mean olive tail moment and mean tail DNA percentages were also measured. (**c**) Representative images of micronuclei formation in mechanically scratched or unscratched confluent skin fibroblasts 24 h following exposure to IR (0–15 Gy). Scale bars represent 20 *μ*m. Cells containing micronuclei were then quantified. (**d**) Flow cytometric analysis using the Annexin V-PI double-staining assay of mechanical scratch or unscratched confluent skin fibroblasts 24 h following exposure to IR (0–50 Gy). **P<0.01; *P<0.05. *P*-values were calculated using the independent-samples *t*-test in **a–d**

DNA DSBs are critical lesions following IR.^7^ *γ*H2AX is a well-known marker for DSBs^8^ that disappears as DNA repair mechanisms cease.^9, 10^ Fibroblasts achieving confluence over 7 days were mechanically scratched 72 h before exposure to 5 Gy IR. *γ*H2AX foci were rapidly formed in the nuclei of scratched and confluent skin fibroblasts, reaching a maximum level 30 min after IR and returning to baseline levels 24 h after IR (Figure 2a). At each time point before recovery (24 h after IR), the number of *γ*H2AX foci was significantly lower in the irradiated scratched cells than in the irradiated confluent cells. To exclude the possibility that the decrease in *γ*H2AX foci formation was simply the result of the mechanical scratch, we compared scratched cells and confluent cells without any IR exposure. Both groups showed low levels of nuclear *γ*H2AX foci formation; however, scratch injury alone slightly increased *γ*H2AX foci formation. Western blot analysis also showed lower levels of *γ*H2AX foci expression and faster decreases in *γ*H2AX expression in irradiated and scratched fibroblasts. However, in tumor cells (A549 and MG63 cells), scratch wounding prior to irradiation had the opposite effect, increasing *γ*H2AX expression and delaying its disappearance (Figure 2a). The presence and repair of DSBs were confirmed by a neutral comet assay that showed significantly shorter olive tail moment and a lower percentage of tail DNA in scratched fibroblasts (Figure 2b).

Further, we evaluated whether mechanical injury could rescue the deleterious effects of IR *in vivo*. For these experiments, mice received full-thickness wounds 3 days before radiation to allow fibroblasts at the wound margins to be fully activated. Wounded and non-wounded normal mice were irradiated at a dose of 6 Gy. *γ*H2AX staining showed that *γ*H2AX-positive fibroblasts showed similar DSBs in normal and wounded skin during the first 2 h after IR. About 4 h later, the population of *γ*H2AX-positive cells and the signal intensity were much lower in wounded fibroblasts (Supplementary Figure S1b). In addition, wounded skin in mice received full-thickness wounds 7 and 14 days before radiation showed similar effects compared to normal skin (data not shown).

### Mechanical injury decreases IR-induced mitotic catastrophe and apoptosis

Mitotic catastrophe (MC) is another principal form of cell damage induced by IR.^11^ We further analyzed whether or not a mechanical scratch could affect IR-induced MC. At 24, 48, and 72 h after IR with doses of 5, 10, and 15 Gy, respectively, scratched and confluent fibroblasts were stained with DAPI and analyzed to determine the incidence of anaphase chromatid bridges and micronuclei phenotype, the two hallmarks of MC. Anaphase chromatid bridges occurred rarely in both scratched and confluent fibroblasts. However, the micronucleus phenotype was remarkably more frequent in confluent cells than in scratched cells (Figure 2c and Supplementary Figure S2). In addition, IR-induced apoptosis of scratched and confluent fibroblasts was investigated using flow cytometry and calcein-AM/PI staining. Both scratched and confluent fibroblasts showed resistance to 5 Gy IR. However, with exposure to high doses of IR (30 and 50 Gy), apoptosis increased in confluent fibroblasts, but not in scratched fibroblasts (Figure 2d and Supplementary Figure S3).

### Mechanical injury heightens DSB repair in human fibroblasts

**Figure 3:**
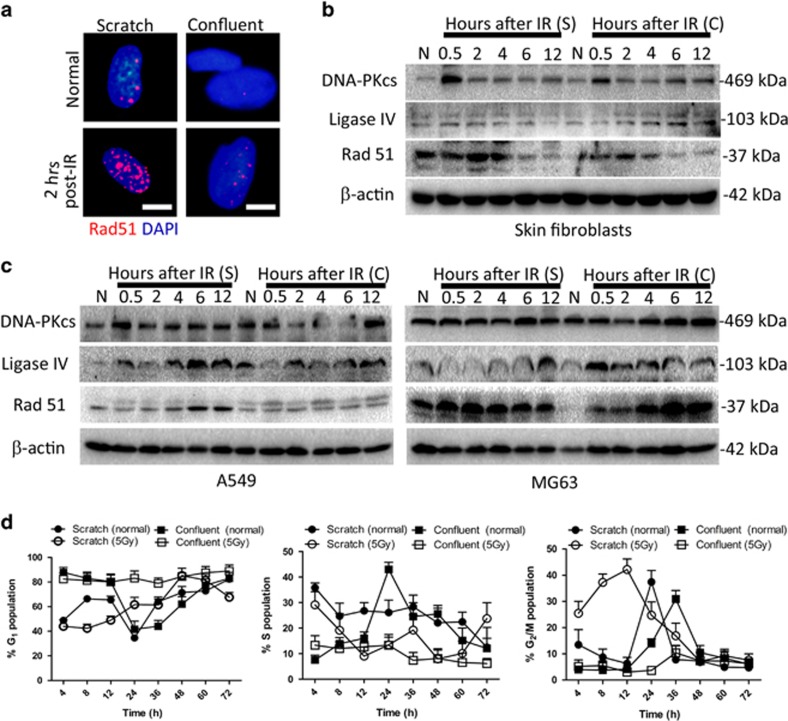
Mechanical injury increases HR repair and accelerates cell cycle arrest recovery of skin fibroblasts. (**a**) Nuclei of mechanically scratched and unscratched confluent skin fibroblasts were co-stained with *Rad51* and DAPI at indicated time points following radiation (5 Gy). Scale bars represent 20 *μ*m. (**b**) Western blot analysis of *DNA-PKcs*, *ligase* IV, and *Rad51* expression in mechanically scratched and unscratched confluent skin fibroblasts following radiation (5 Gy). N, normal; S, scratch; C, confluent. (**c**) Same as in **b**, but cells are A549 and MG63 cells. (**d**) Average fraction (%) of mechanically scratched and unscratched confluent skin fibroblasts in G1, S, and G2/M phase at indicated time points following radiation (0 or 5 Gy)

To test the DSB repair mechanisms in scratched fibroblasts following IR, we investigated the expression of ligase IV, DNA-PKcs (NHEJ-related proteins), and Rad51 (an HR-related protein) in scratched and confluent fibroblasts following IR. While the expression of Rad51 was significantly higher in scratched fibroblasts than in confluent fibroblasts (Figures 3a and b), the expression of DNA-PKcs and ligase IV did not differ significantly (Figure 3b). We also compared the expression of ligase IV, DNA-PKcs, and Rad51 in non-irradiated scratched cells and confluent cells and found that Rad51 was upregulated following mechanical injury (Figures 3a and b). These results indicated that mechanical injury enhanced the HR repair of fibroblasts. However, in tumor cells, the increase in Rad51 expression was not as notable in scratched cells as it was in fibroblasts (Figure 3c).

### Mechanical injury accelerates human fibroblasts recovery from IR-induced cell cycle arrest

The increases in cell survival and colony size after IR in scratched fibroblasts suggested that mechanical injury was affecting the activation of DNA damage checkpoints. Consistent with previous studies,^12^ most confluent fibroblasts remained in the G1 phase under normal conditions. After reseeding, they quickly entered the S phase, reaching a peak 24 h later, and then entered into G2/M phase a subsequent 12 h later. Altogether, the full cell cycle in human fibroblasts took 36 h (Figure 3d and Supplementary Table S1). We observed that after exposure to 5 Gy IR, confluent fibroblasts arrested mainly at the G1/S phase with recovery occurring 24 h after IR, while scratched fibroblast arrested mainly at the G2/M phase with recovery occurring 12 h after IR (Figure 3d and Supplementary Table S1). These results indicated that mechanical injury accelerated the recovery of fibroblasts from cell cycle arrest induced by IR.

### Mechanical injury induces the stemness phenotype change in human fibroblasts

**Figure 4:**
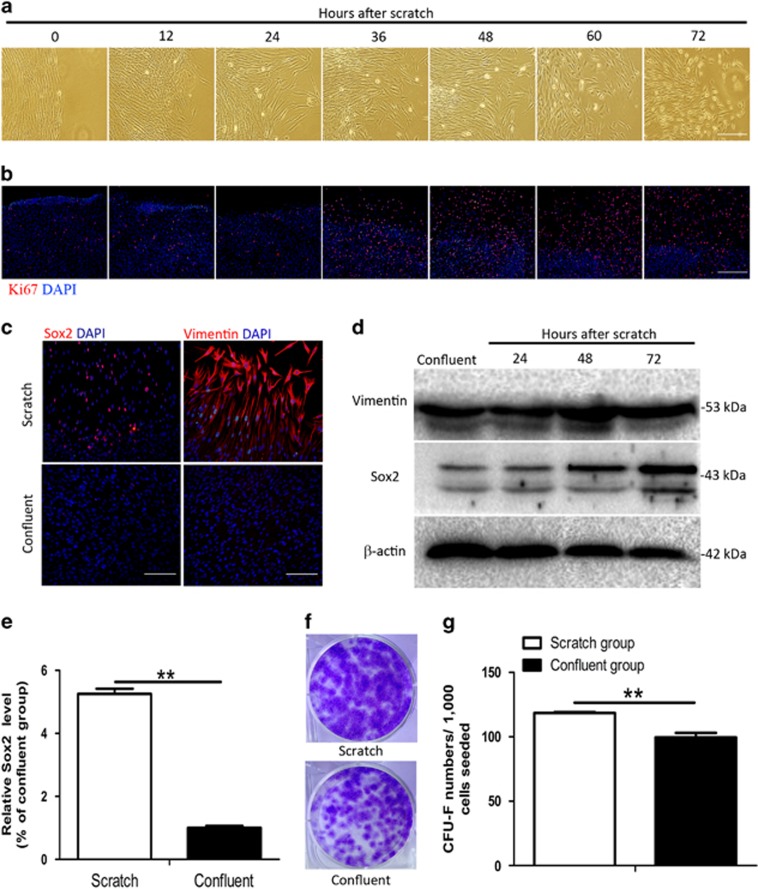
Characterization of changes in skin fibroblasts following mechanical injury. (**a**) Representative images of skin fibroblasts in wound margins following mechanical scratch. Scales bars represent 500 *μ*m. (**b**) Immunofluorescence staining for *Ki67* demonstrating activation of skin fibroblasts following mechanical scratch. Cell nuclei are counterstained with DAPI. Scales bars represent 500 *μ*m. (**c**) Immunofluorescence staining for *Sox2* and *vimentin* in skin fibroblasts at the scratch edge 72 h after mechanical scratch. Cell nuclei are counterstained with DAPI. Scales bars represent 200 *μ*m. (**d**) Western blot analysis of *Sox2* and vimentin expression at indicated time points in skin fibroblasts following mechanical scratch. (**e**) Real-time PCR analysis of *Sox2* expression in skin fibroblasts 72 h after mechanical scratch. (**f**) Colony formation assay of skin fibroblasts 72 h after mechanical scratch. (**g**) Quantification of colonies in **f**. ^**^P<0.01. *P*-values were calculated using the independent-samples *t*-test in **e** and **g**

To determine how mechanical injury could increase the radioresistance of human fibroblasts, we further investigated cell phenotype changes after wounding. After scratching, fibroblasts quickly migrated into the wound within 1 h. 24 h later, the cells at the wound margin developed a small, round phenotype, which peaked in prevalence 72 h later (Figure 4a). In addition, Ki67 staining showed that the proliferation of fibroblasts in the wound margin was completely activated 36 h after scratching (Figure 4b). However, in tumor cells, proliferation was only partially inhibited by confluence, and activation of proliferation occurred sooner (12 h) after mechanical injury (Supplementary Figure S4). Further experiments revealed that the activation of proliferation in fibroblasts was not dependent on wound size, either *in vitro* or *in vivo* (Supplementary Figures S5 and S6).

Molecular markers of fibroblast activation were analyzed to further understand phenotype changes in fibroblasts in response to wounding. Given recent studies have associated the stemness phenotype with radioresistance in some stem cell types,^13^ markers of stemness in fibroblasts were specifically investigated. Expression of the pluripotent stem cell marker sex-determining region Y-box 2 (Sox2) and the mesenchymal cell marker vimentin was upregulated at wound margins (Figure 4c). Other tested markers, including octamer-binding transcription factor-4 (Oct4), Nanog, collagen I, chemokine receptor-4 (CXCR4), fibronectin, and *α*-smooth muscle actin (*α*-SMA), were unchanged after scratching (Supplementary Figure S7). We then determined the spatial and temporal changes in Sox2 and vimentin expression. Vimentin was strongly expressed in migratory cells in the cavity of the wound 12 h after scratching and returned to normal levels after the wound healed. Expression of Sox2 began to increase at 48 h after scratching and reached a peak at 72 h (Figures 4d and e). The expression of Sox2 and vimentin increased in nearly all fibroblasts at wound margins (Supplementary Fig. S8*a*). Nearly all cells strongly expressing Ki67 stained with high intensity for Sox2 (Supplementary Figure S8b), indicating that Sox2 might be associated with the proliferation of fibroblasts in response to injury.

### Mechanical injury induces changes in the transcriptional state of human fibroblasts

**Figure 5:**
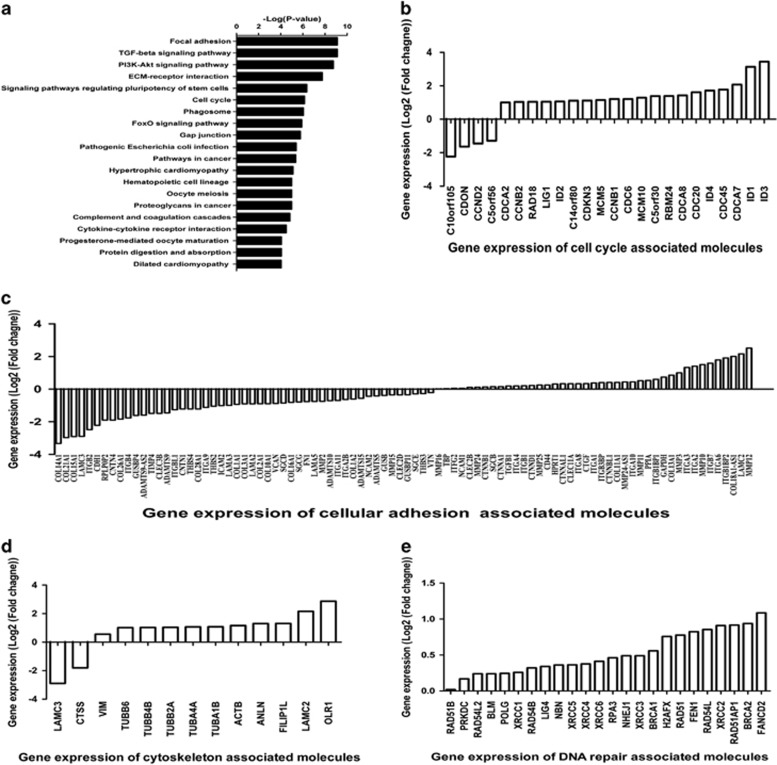
Transcriptional changes in skin fibroblasts following mechanical injury. Skin fibroblast monolayers were grown to confluence over 7 days and scratched. Total RNA of mechanically scratched and unscratched confluent skin fibroblasts were harvested 72 h later. Expression profiles were analyzed. (**a**) KEGG pathway analyses of transcriptomes of mechanically scratched and unscratched confluent skin fibroblasts. (**b–e**) Altered expression of genes encoding proteins associated with the cell cycle, cellular adhesion, cytoskeleton, and DNA repair was observed

To further explore enhanced radioresistance induced by mechanical injury, gene expression profiling coupled with bioinformatic analyses were performed and revealed that the focal adhesion pathway, TGF-*β* signaling pathway, and PI3K-Akt signaling pathway were the three most differentially modulated canonical pathways, following mechanical scratch wounding (Figure 5a). There was significant upregulation of genes involved in the cell cycle and activation of proliferation (Figure 5b), consistent with previous data demonstrating high levels of Ki67 expression in response to mechanical injury. The expression of cell adhesion-associated genes also changed greatly following mechanical injury (Figure 5c), suggesting that elements of the cell membrane responsible for cell adhesion and communication likely play an important role in the sensing of mechanical injury. In addition, our results were consistent with those of previous studies^14, 15, 16^ showing that the transforming growth factor-*β* (TGF-*β*) signaling pathway is intimately involved in wound healing. Transcriptome analysis also demonstrated significantly increased expression of cytoskeleton molecules (Figure 5d), consistent with observations of fibroblast migration from the wound margins directly into the wound cavity after mechanical injury. Furthermore, gene expression of DNA DSB repair-related proteins, particularly the HR system components such as Rad51, XRCC2, and BRCA2, were also upregulated following scratch wounding (Figure 5e). However, expression of anti-apoptotic genes such as*bcl2*or*bcl-xl* showed no significant changes. Taken together, these transcriptional changes provided further evidence at the genomic level of enhanced radioresistance in fibroblasts after mechanical injury.

### PI3K/Akt pathway activation involves fibroblast-sensing of mechanical injury

**Figure 6:**
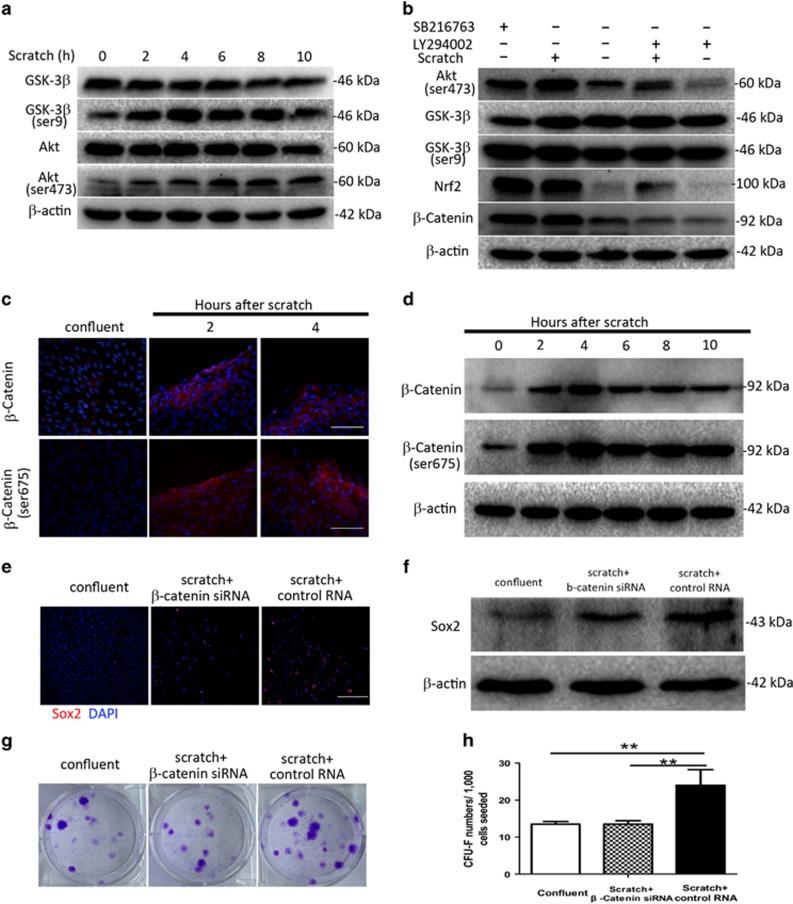
Mechanical injury activates the *PI3K/Akt* pathway, and *β-catenin* enhances stemness of skin fibroblasts. (**a**) Western blot analysis of total *GSK-3β*, phospho-*GSK-3β* at Ser-9, and upstream *Akt* (total and phosphorylated at Ser-473) in skin fibroblasts following mechanical scratch. (**b**) Western blot analysis of skin fibroblasts treated with LY294002 and SB216763. (**c**) Immunofluorescence staining of total *β-catenin* and phospho-*β-catenin* at Ser-675 at 2 and 4 h post scratch. Scale bars represent 200 *μ*m. (**d**) Western blot analysis of total *β-catenin* and phospho-*β-catenin* at Ser-675 at various time points following mechanical scratch. (**e** and **f**) Immunofluorescence staining and western blot analysis of *Sox2*, indicating a decrease in expression following *β*-*catenin* knockdown. (**g**) Clonogenic survival assay of mechanically scratched and unscratched confluent skin fibroblasts following knockdown of *β-catenin* when exposing to radiation of 5 Gy. (**h**) Quantification of colonies in **g**. **P<0.01. *P-*values were calculated using the one-way analysis of variance

The transcriptional expression data showed that both the focal adhesion and PI3K/Akt pathways were activated following mechanical injury. Previous studies have shown that focal adhesion complexes sense mechanical signals, activating inner focal adhesion kinase (FAK) through focal adhesion complexes^17, 18, 19^ that in turn activate the PI3K/Akt pathway.^20, 21^ The PI3K/Akt pathway is involved in a range of biological processes, including cell survival, proliferation, migration, and stem cell self-renewal.^22^ To explore the role of the PI3K/Akt pathway in fibroblast radioresistance following mechanical injury, we investigated the expression of total Akt and activated serine 473 (ser-473) phosphorylated Akt following mechanical wounding. Ser-473-phosphorylated Akt increased 2 h after scratching (Figure 6a). GSK-3*β*, a serine/threonine kinase involved in the sensing of mechanical signals,^23, 24, 25^ is inactivated by phosphorylation of Serine-9 (ser-9). Our data also showed that the expression of ser-9 phosphorylated GSK-3*β* increased at the same time as upregulation of ser-473 phosphorylated Akt (Figure 6a). The expression of *β*-catenin and Nrf2, two downstream proteins of GSK-3*β,*^26, 27^ increased with the inactivation of GSK-3*β* following mechanical injury (Figure 6b). The administration of LY294002, a PI3K inhibitor, decreased the expression of ser-473-phosphorylated Akt and ser-9-phosphorylated GSK-3*β* and abolished scratch-induced upregulation of *β*-catenin and Nrf2, further verifying that the PI3K/Akt pathway was being activated by mechanical injury. Inactivation of GSK-3*β* by the inhibitor SB216763 also significantly increased the expression of *β*-catenin and Nrf2 in confluent fibroblasts (Figure 6b). These results indicated that mechanical injury upregulates the expression of *β*-catenin and Nrf2, two critical transcription factors involved in cell proliferation and survival, by activating the PI3K/Akt pathway.

### *β*-catenin initiates conversion to the stemness phenotype in human fibroblasts following mechanical injury

According to our gene expression data, the focal adhesion pathway is primarily responsible for sensing mechanical injury. In addition to focal adhesion complexes, cadherin–cadherin interactions between cells are also critical sensors of mechanical injury. *β*-catenin is a ubiquitous transcription factor located on the inner surface of cadherins that can be activated at the cell membrane, resulting in its nuclear translocation and the transcription of several genes related to proliferation and stemness.^28^ Confirming our earlier data showing increased transcriptional levels of *β*-catenin in scratched fibroblasts, immunofluorescence staining demonstrated that both total *β*-catenin and activated (phosphorylated at Ser-675) *β*-catenin levels were higher in fibroblasts at the margins of the wound cavity (Figure 6c). Western blot analysis also showed that the expression of total and activated *β*-catenin was elevated 2 h following scratching (Figure 6d). To further elucidate the role of *β*-catenin in the radioresistance of fibroblasts, siRNA knockdown of *β*-catenin was performed (Supplementary Figure S9). Knockdown of *β*-catenin resulted in minimally decreased expression of vimentin following scratching (Supplementary Figure S10), but significantly decreased the expression of Sox2 (Figures 6e and f). Furthermore, the survival of fibroblasts after IR was decreased by *β*-catenin knockdown (Figures 6g and h). These results indicated that *β*-catenin plays a critical role in the conversion to a stemness phenotype in fibroblasts following mechanical injury and contributes to their radioresistance.

### Nrf2 mediates increased antioxidant capability and DSB repair in human fibroblasts following mechanical injury

**Figure 7:**
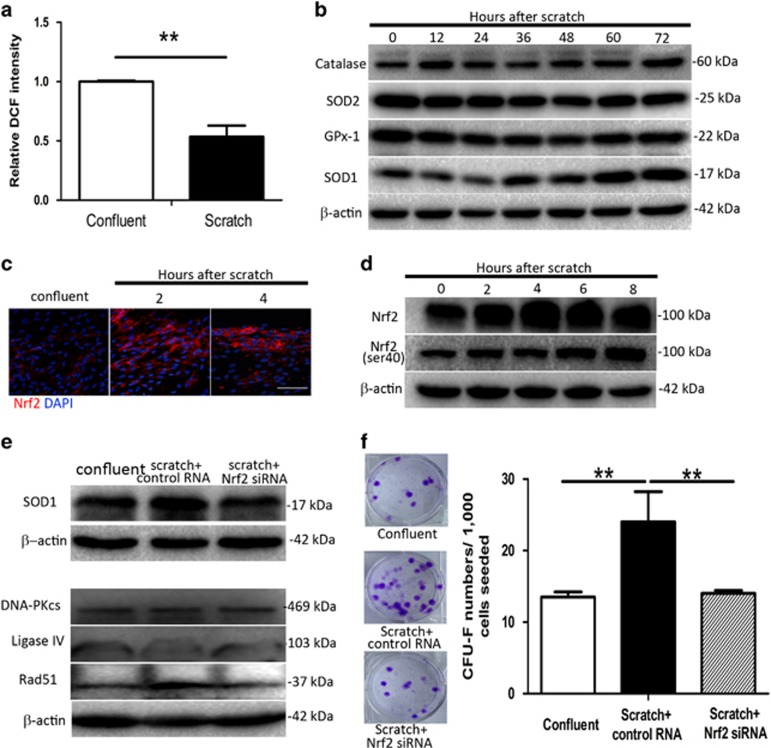
*Nrf2* mediates mechanical injury-induced enhancement of antioxidant ability and DSB repair in skin fibroblasts. (**a**) DCFH-DA flow cytometric analysis of endogenous ROS levels in skin fibroblasts 72 h following mechanical scratch. (**b**) Western blot analysis of antioxidant proteins in skin fibroblasts at indicated time points following mechanical scratch. (**c**) Immunofluorescence staining for total *Nrf2* at 2 and 4 h post scratch. Scales bars represent 200 *μ*m. (**d**) Western blot analysis of total *Nrf2* and phospho-*Nrf2* at Ser-40 in skin fibroblasts following mechanical scratch. (**e**) Western blot analysis of *SOD1* and DNA repair-related proteins following knockdown of *Nrf2*. (**f**) Post-radiation clonogenic survival assay of mechanically scratched and unscratched confluent skin fibroblasts following knockdown of *Nrf2*. **P<0.01. *P-*values were calculated using the independent-samples *t*-test in **a** and the one-way analysis of variance in **f**

Reactive oxygen species (ROS) are critical mediators of IR-induced cell damage. Our observation that *γ*H2AX foci formation decreased and resolved more quickly in response to mechanical injury suggested an increase in the antioxidant activity and DNA repair in fibroblasts following mechanical injury. Endogenous ROS levels were observed to decrease following mechanical injury (Figure 7a). We tested the expression of several well-known antioxidant enzymes, including superoxide dismutase1 (SOD1), SOD2, catalase, and glutathione peroxidase 1 (GPx-1), and found that SOD1 expression increased significantly following scratching (Figure 7b). The upregulation of SOD1 in scratched fibroblasts did not occur immediately after scratch injury but 36 h later, demonstrating that the upregulation of SOD1 is a secondary molecular event triggered by mechanical injury. Previous studies have indicated that Nrf2 is a key regulator of antioxidant gene expression.^29^ The above data have indicated that the increases of Nrf2 in fibroblasts resulted from the inactivation of GSK-3*β*. Immunofluorescence staining also showed enhanced Nrf2 expression in fibroblasts at the margins of the wound cavity (Figure 7c), and western blot analysis revealed an increase in both total Nrf2 and activated (phosphorylated at serine 40) Nrf2 proteins (Figure 7d). However, expression of antioxidant proteins did not increase following mechanical injury in A549 and MG63 tumor cells (Supplementary Figure S11). In further support of Nrf2-mediated regulation of antioxidant gene expression, siRNA knockdown of Nrf2 (Supplementary Figure S9b) diminished mechanical injury-induced upregulation of SOD1 (Figure 7e). Rad51 expression was also diminished by knockdown of Nrf2, while ligase IV and DNA-PKcs were not affected (Figure 7e). Fibroblast survival following IR was also significantly decreased by Nrf2 knockdown (Figure 7f). Taken together, these findings indicated that Nrf2 is upregulated via the PI3K/Akt pathway following mechanical injury, which in turn increases cellular antioxidant capability and DNA DSB repair by upregulating SOD1 and Rad51 expression, respectively. These changes collectively contribute to increased radioresistance in fibroblasts after mechanical injury.

## Discussion

**Figure 8:**
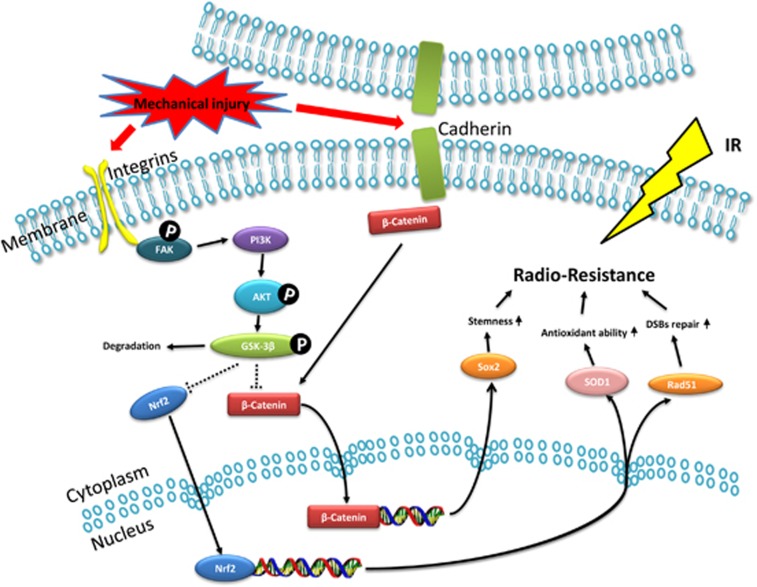
Schematic illustration of mechanical injury-induced increase of radiation resistance in skin fibroblasts. Following mechanical injury, cadherin–cadherin interactions are disrupted, and *β-catenin* dissociates from membrane complexes. Focal adhesion complexes mediate mechanotransduction of mechanical injury signals into intracellular chemical signals by activating *FAK*. The downstream *PI3K/Akt* pathway is then activated. Following *GSK-3β* is phosphorylated at Serine-9 by Akt activation, which contributes to the degradation of *GSK-3β*. Thus, the *GSK-3β*-targeted proteins *Nrf2* and *β-catenin* accumulate and are translocated into the nucleus, where they promote stemness, antioxidant ability, and DSB repair of skin fibroblasts through activation of related genes such as *Sox2*, *SOD1*, and *Rad51*, increasing radioresistance

Using complementary cellular, molecular, and genomic approaches, we have discovered that mechanical injury increases the radioresistance of skin fibroblasts by increasing the stemness, antioxidant capability, and mechanisms of DNA DSB repair (Figure 8). Following mechanical injury, cadherin–catenin complexes are interrupted, and *β*-catenin is activated at the membrane.^30^ In addition, focal adhesion complexes can sense mechanical signals that activate the inner FAK,^17, 18, 19^ which in turn activates the PI3K/Akt pathway.^20, 21^ GSK-3*β*, a serine/threonine kinase involved in a diverse range of signaling pathways, mediates crosstalk between the PI3K/Akt and *β*-catenin pathways after mechanical injury. Although previous studies have shown that GSK-3*β* can mediate *β*-catenin^23, 31^ or Nrf2,^27^ the regulation of both Nrf2 and *β*-catenin at the same time by GSK-3*β* in response to a single stimulation factor has not been reported until now.

The radioresistance of stem cells has received much recent attention.^13, 32^ Upon mechanical injury, skin fibroblasts change to a relatively primitive differentiation state via a *β*-catenin-dependent pathway. Other studies have also reported that scratch injury or conditioned culture medium from scratch-insulted astrocytes induces de-differentiation of astrocytes into a neural progenitor phenotype.^30, 33^ In a previous study, we demonstrated that wound trauma-mediated stemness phenotype changes in mouse skin fibroblasts *in vivo.*^34^ In this study, we observed that the change in stemness phenotype increased the radioresistance of human skin fibroblasts. Similarly, it has been shown that inducing astrocyte differentiation into neural progenitor phenotypes also contributes to increased radioresistance.^35^ In contrast, mesenchymal stem cells, induced to differentiate, exhibited sharply prolonged DNA DSBs and increased cell death after IR, even during the early steps of differentiation.^36^ However, why stem cells exhibit increased radioresistance remains unclear. According to a study by Mohrin *et al.,*^37^ the resistance of hematopoietic stem cells to the genotoxic stresses of IR was independent of quiescence. Induction of cell proliferation by G-CSF did not decrease their radioresistance and did not enhance susceptibility to acquisition of genetic mutations. Inducing changes in the stemness of cells remains a possible approach to induce radioresistance; however, further studies are needed to clarify the mechanisms underlying this process.

Enhanced antioxidant capability and DNA damage repair is another key consideration for enhancing radioresistance.^38^ Nrf2 is a critical transcriptional factor that controls the expression and coordinated induction of a battery of genes that encode biotransformation reactions, redox homeostasis, energetic metabolism, DNA repair, and proteostasis.^39, 40^ The activation of Nrf2 often results from chemical or oxidative stress and causes the dissociation of Nrf2 from Keap1. However, our study is the first to report that mechanical injuries can directly induce upregulation of Nfr2. Nrf2 further regulates the expression of SOD1 in fibroblasts to eliminate the excessive ROS produced directly from ionized water by ionizing particles^41^ or indirectly generated by damaged mitochondria^42, 43^ following IR exposure.^44, 45^ Other studies have also reported that directly or indirectly upregulated antioxidant proteins protect cells from IR-induced damage.^46, 47^ In addition, the increase in Nrf2 expression following mechanical injury increases DNA DSB repair by upregulating Rad51 expression. A recent study has also shown that Nrf2 increases the radioresistance of cancer cells by targeting Rad51 expression in a ROS-independent manner.^48^ According to Kim *et al.*,^49^ Nrf2 regulates the expression of 53BP1 to protect colonic epithelial cells from ionizing radiation.^49^ In summary, Nrf2 may be another potential mediator for radioresistance by regulating both antioxidant capability and DNA damage repair.

Lastly, the findings in the present study may have important and direct clinical relevance. Adjuvant radiation after surgery is currently a standard of care treatment for multiple cancer types (PMID: 25468225, PMID: 24625455, PMID: 25366825, PMID: 21946673). Although the exact timing of radiotherapy in the postoperative period is debatable, conventional wisdom has dictated that earlier radiotherapy, where tumor volume is maximally low, may maximize its efficacy. On the other hand, insufficient time between initial surgery and radiation is presumed to complicate wound healing related to radiating normal tissues mechanically disrupted by surgery. Importantly, our study demonstrates that mechanical injury may in fact confer radioresistance to normal human skin fibroblasts, while radiosensitizing tumor cells. Our *in vitro* and *in vivo* data suggest that the cellular and molecular changes associated with radioresistance may be maximally induced within 72 h after mechanical injury. These findings raise the possibility that radiation may be administered within days after surgery in the clinical setting and may, furthermore, improve rates of wound-healing complications, compared to the standard timing delay of adjuvant radiotherapy. Likewise, earlier radiotherapy may also capitalize upon the radiosensitizing effects of mechanical injury in tumor tissues and lead to an enhanced reduction of overall tumor burden. Further studies may ultimately determine whether these unconventional findings can be translated into changes of clinical practice. In addition, the roles of the enhanced radioresistance and the surviving fibroblasts in wound healing following radiation combined injuries and radiation therapy-associated chronic side effects still needed further study.

## Materials and methods

### Animals

C57/BL mice were obtained from the Center for Experimental Animals at the Third Military Medicine University (TMMU, Chongqing, China). *In vivo* experiments were conducted in accordance with the Guidelines for the Care and Use of Laboratory Animals of the TMMU, and all procedures were approved by the Animal Care and Use Committee of the TMMU.

### Combined radiation and skin wound model

To investigate tissue expression of *γ*H2AX, mice in the IR alone group were irradiated at a dose of 6 Gy, and the dorsal skin tissues were obtained at 0.5, 2, 4, 6, and 12 h after IR. Circular, full-thickness skin samples 1 cm in diameter were surgically excised from the middle back of mice in the post-wound IR group. Three days later, the wounded mice were irradiated at a dose of 6 Gy, and the wound tissues were obtained 0.5, 2, 4, 6, and 12 h after IR. Paraffin sections were stained using rabbit anti-*γ*H2AX (1:200; #9718, Cell Signaling, Danvers, MA, USA), and nuclei were co-stained with DAPI (Beyotime, Haimen City, Shanghai, China).

To isolate granulation-derived cells, mice in the pre-wound IR group were irradiated at a dose of 6 Gy, and the skin was wounded immediately after IR. Mice in the post-wound IR group were wounded 3 days before 6 Gy radiation. Granulation tissues were harvested from mice in the two groups 10 days after wounding.

### Cell isolation and culture

Primary human fibroblasts were obtained after circumcision of foreskins and approval of the protocol by the ethics committee of TMMU. Isolation protocols have been previously described.^50^ In brief, foreskin tissues were cut into 1–2 cm^2^ pieces after subcutaneous tissue removal and digested for 1 h at 37 °C in a digestion medium containing 1 mg/ml dispase (Roche, Basel, Switzerland). The epidermis was then stripped and digested for another 1 h at 37 °C in the digestion medium consisting of DMEM with 0.25% collagenase I (Worthington Biochemical, Beijing, China). The digested cells were then passed through a 75-*μ*m cell strainer, centrifuged, and re-suspended in Iscove's modified Dulbecco's Medium (HyClone, Logan, UT, USA) supplemented with 10% fetal bovine serum (Gibco, Carlsbad, CA, USA), 100 U/ml penicillin, and 0.1 mg/ml streptomycin (all products obtained from Beyotime). Cells were grown to >90% confluence, trypsinized (trypsin-EDTA 0.5% w/v, Hyclone), and re-plated for experiments.

Mouse granulation tissue cells were isolated as previously described.^34^ Briefly, granulation tissues were harvested 10 days after wounding and cut into 1 cm^2^ pieces and digested for 1 h at 37 °C in a digestion medium containing 0.25% collagenase I (Worthington Biochemical). The following protocols and culture conditions were the same as those used for human fibroblast isolation and culture. Tumor cell lines (A549 and MG63) were purchased from ATCC (CCL-185 and CRL-1427) and cultured under the same conditions as those for human fibroblasts. LY294002 (50 *μ*M; Sigma, St. Louis, MO, USA) and SB216763 (10 *μ*M; Sigma) were used to inhibit PI3K and GSK-3*β*, respectively.

### Scratching of monolayers

Monolayers of fibroblasts or tumor cells grown to 100% confluence over a period of 7–14 days were scratched using a sterile pipette tip. For immunofluorescence experiments, monolayers on chamber slides were scratched once linearly with a 200-*μ*l pipette tip. For other experiments, monolayers on a six-well plate were scratched with a 1000-*μ*l pipette tip in an 8 × 8 grid pattern; monolayers on 10-cm diameter Petri dishes were scratched with a 1000-*μ*l pipette tip in a 20 × 20 grid pattern. In all cases, non-adherent cells were removed immediately after scratching by changing the culture medium.

### Irradiation

Cells were irradiated in an X-ray irradiator (RS2000; Rad Source Technologies, Alpharetta, GA, USA) at room temperature with an absorption rate of 1.284 Gy/min. A dose of 5 Gy was administered. Animals received total body irradiation at a dose of 6 Gy.

### Statistical analysis

Statistical analyses were performed using the SPSS 13.0 package (SPSS Inc., Chicago, IL, USA). Results were expressed as the mean±S.D. An independent-samples *t*-test was used to determine significant differences between two groups. Comparisons of multiple groups were performed using one-way analysis of variance with corrections for multiple comparisons. *P*<0.05 was considered to be statistically significant.
