# Supplementary material for: Increase in the radioresistance of normal skin fibroblasts but not tumor cells by mechanical injury
Source: Cell Death Dis. 2017 Feb 2;8(2):e2573–. doi: 10.1038/cddis.2016.416 (PMC5386452; doi:10.1038/cddis.2016.416)
Supplement: Supplementary Information [file cddis2016416x1.docx]

**Supplementary Materials**

**Supplementary methods**

***Immunoﬂuorescence and immunohistochemistry***

Immunoﬂuorescence and immunohistochemistry were performed as described previously ^34^. The following were used as primary antibodies: rabbit anti-Sox2 (1:200, ab97959, Abcam, Cambridge, UK), mouse anti-Sox2 (1:200, MAB2018, R&D Systems, Minneapolis, MN, USA), rabbit anti-Vimentin (1:200, bs-0756R, Bioss Inc., Woburn, MA, USA), rabbit anti-γH2AX (1:200, #9718, Cell signaling), rabbit anti-Rad51 (1:100, sc-8349, Santa Cruz Biotechnology, Dallas, TX, USA), rabbit anti-Ki67 (1:200, ab15580, Abcam), rabbit anti-β-catenin (1:200, ab6302, Abcam), rabbit anti-β-catenin (ser675) (1:200, #9567, Cell signaling), rabbit anti-Nrf2 (1:200, ab62352, Abcam), rabbit anti-Nrf2 (Ser40) (1:200, ab76026, Abcam), rabbit anti-collagen I (1:200, ab34710, Abcam), mouse anti-α-SMA (1:500, A5228, Sigma). Incubations were performed at 4°C overnight and detections were performed with a secondary donkey anti-mouse or rabbit IgG-Cy3, Alexa Fluor 488, or horseradish peroxidise (HRP) antibody (all obtained from Beyotime). Cells were counterstained with the nuclear dye 4′,6-diamidino-2-phenylindole (Beyotime) and examined using a fluorescence microscope. Hematoxylin stain was used for immunohistochemistry.

***Western blot analysis***

Total proteins from fibroblasts were extracted using cell lysis buffer (Beyotime) supplemented with proteinase inhibitor cocktail (Roche). After vortexing and centrifugation at 12000 *×g* for 10 min at 4°C, the supernatant was collected and protein was quantitated using a BCA protein assay kit (Beyotime). Equal amounts of protein for each sample were subjected to 8–12% SDS-PAGE and transferred to polyvinylidene diﬂuoride membranes (Millipore, Darmstadt, Germany). Membranes were blocked in blocking buffer (Beyotime) for 1 hour before application of primary antibodies. The following were used as primary antibodies: γH2AX, Sox2, vimentin, Rad51, β-catenin, β-catenin (s675), Nrf2, Nrf2 (s40,) (1:1000; manufacturers as above), DNA PKcs, ligase IV, SOD1, SOD2, Catalase, Glutathione Peroxidase 1 (1:1000, ab80514, ab70250, ab16831, ab13533, ab16731 and ab22604, Abcam), GSK-3β, GSK-3β (ser9) (1:1000, #9315 and #9322, Cell signaling), Akt, Akt (ser473) 1:1000, #9916, Cell signaling), β-actin (1:1,000, AA128, Beyotime). HRP-conjugated horseradish goat anti-mouse or anti-rabbit secondary antibody (1:1,000, A0208 and A0216, Beyotime) was applied for 2 h at room temperature. Blots were visualized using chemiluminescence (Bio-Rad, Hercules, CA, USA).

***RNA sequencing and quantitative real-time PCR analysis***

Total RNA was extracted using RNAiso Plus (TaKaRa/Clontech, Mountain View, CA, USA). RNA sequencing and bioinformatic analyses were performed by Ribobio Co (Guangdong, China). Quantitative PCR was conducted using a SYBR Premix Ex TaqII (Tli RNaseH Plus) real-time PCR kit (TaKaRa/Clontech) according to the manufacturer’s protocol. Relative expression was normalized to the expression of GAPDH. The primers are as follows: SOX2, F:GGGAAATGGGAGGGGTGCAAAAGAGG, R: TTGCGTGAGTGTGGATGGGATTGGTG; GAPDH, F: AGGTCGGTGTGAACGGATTTG, R: TGTAGACCATGTAGTTGAGGTCA. Three independent experiments were performed. In each experiment, triplicate procedures were performed for each group.

***Colony-forming unit fibroblast assay***

For clonogenic survival assays after IR, human fibroblasts were grown to 100% conﬂuence over 7 days and scratched. Seventy-two hours later, scratched and confluent cells were irradiated at a dose of 0–7 Gy and seeded onto 6-well plates at a density of 1000 cells per well on 6-well plates. Cells were cultured for 12 days. Colonies were stained with crystal violet and counted as previously described ^34^. To test colony-forming ability of mouse granulation tissue-derived cells, cells were seeded at a density of 1500 cells per well in 6-well plates. Three independent experiments were performed for each assay.

***Measurement of mitotic catastrophe***

Human fibroblasts were grown to 100% conﬂuence over 7 days and scratched. Seventy-two hours later, scratched and confluent cells were irradiated at doses of 5, 10, and 15 Gy following reseeding at a density of 1 × 10^5^ cells/cm^2^. At 24, 48, and 72 hours following radiation, cells were fixed and stained with DAPI. The anaphase chromatid bridges and micronuclei phenotypes were then captured with a fluorescence microscope. An average of 400 cells in interphase and 200 cells in mitosis from 3 independent experiments were counted.

***Cell cycle analysis***

Fibroblasts cultured in 6-well plates were grown to 100% conﬂuence over 7 days and scratched. Seventy-two hours later, scratched and confluent cells were treated with 5 Gy IR. Irradiated or non-irradiated normal cells were then reseeded at a density of 10^5^/cm^2^. Cells were collected at the indicated time points and fixed in 70% ethanol overnight, followed by PBS washes and staining with propidium iodide (50 µg/mL; Sigma) for 20 min at room temperature. Samples were measured on a BD Accuri C6 Flow Cytometer and analyzed with FlowJo 7.6.1 software (TreeStar Inc., Ashland, OR, USA). Three independent experiments were performed for each group.

***Analysis of apoptosis using Annexin-V–PI staining***

Irradiated confluent or scratched cells (72 hours after scratching) were harvested at the indicated time points and then re-suspended in Annexin-V binding buffer (10 mM HEPES, 150 mM NaCl, 5 mM KCl, 1 mM MgCl_2_, and 1.8 mM CaCl_2_, pH 7.4) with FITC-conjugated Annexin-V (5 µL per test; BD Biosciences, San Jose, CA, USA) and propidium iodide (PI) (50 µg/mL; Sigma) for 20 min. Samples were analyzed using a BD Accuri C6 Flow Cytometer. Three independent experiments were performed for each group.

***Cell live-dead assay***

Irradiated confluent or scratched cells (72 hours after scratching) or positive control (H_2_O_2_, 1mM for 6 hours) were co-stained with Calcein-AM (2 umol/L, Invitrogen) and PI (4umol/L) solution according to manufacturer’s protocol. After rinsed twice with PBS, live cells stained by Calcein-AM and dead/late apoptosis cells stained by PI were visualized with inverted fluorescent microscope (Olympus IX70).

***Measuring endogenous ROS levels***

Cells were harvested at a density of 5–10 × 10^5^/mL, incubated with 2′,7′-dichlorofluorescin diacetate (1:1000; Beyotime) for 20 min at 37°C. The levels of intracellular ROS were determined by measuring the mean fluorescence intensity of 2′,7′-dichlorofluorescein using a BD Accuri C6 Flow Cytometer. Three independent experiments were performed in triplicate for each group.

***Neutral comet assay***

Fibroblasts were grown to 100% conﬂuence in 6-well tissue culture plates over 7 days. Two wells per time point were scratched, and the medium was changed in all wells. Cells were irradiated 72 hours later at a dose of 5 Gy. At the indicated time points, cells were trypsinized, re-suspended at a concentration of 1 × 10^5^ cells/mL of ice-cold PBS and then mixed at a 1:10 ratio (v/v) with low-melting-point agarose (Trevigen, Gaithersburg, MD, USA). Cell suspensions were placed on slides, and the agarose was allowed to cool for 30 min at 4°C in the dark. Cells on slides were then lysed for 1 h at 4°C in the dark in lysis buffer. Slides were immersed with cold neutral electrophoresis buffer for 30 min and placed in a horizontal electrophoresis chamber. A voltage of 22V (1.0 V/cm) was applied for 45 min at 4°C. Slides were immersed in DNA precipitation solution for 30 min in the dark at room temperature, dehydrated in 70% ethanol for 30 min, and were allowed to dry at 37°C for 10–15min in the dark. Samples were then stained with ethidium bromide (Sigma) and visualized immediately. Comets were visualized using a ﬂuorescence microscope and analyzed using open-source CASPlab software (www.casplab.com) with over 50 comets/group at each time point.

***RNA interference***

After fibroblasts were grown to 80% confluence, siRNA oligomers (50nM) against endogenous β-catenin (R: CUGCGGAAGAUGGGAUCAATT; F: UUGAUCCCAUCUUCCGCAGTT) and Nrf2 (R: CCUGCUACUUUAAGCCAUUTT; F: AAUGGCUUAAAGUAGCAGGTT) were transfected with Lipofectamine2000 (Invitrogen, Carlsbad, CA, USA). The interference rate was measured 72 hours later by western blot on transfected lysates and remaining monolayers of fibroblasts were grown to confluence and then scratched as described above. Clonogenic survival following IR, protein expression patterns, and stem-cell markers were subsequently investigated.

**SI Figures**

**
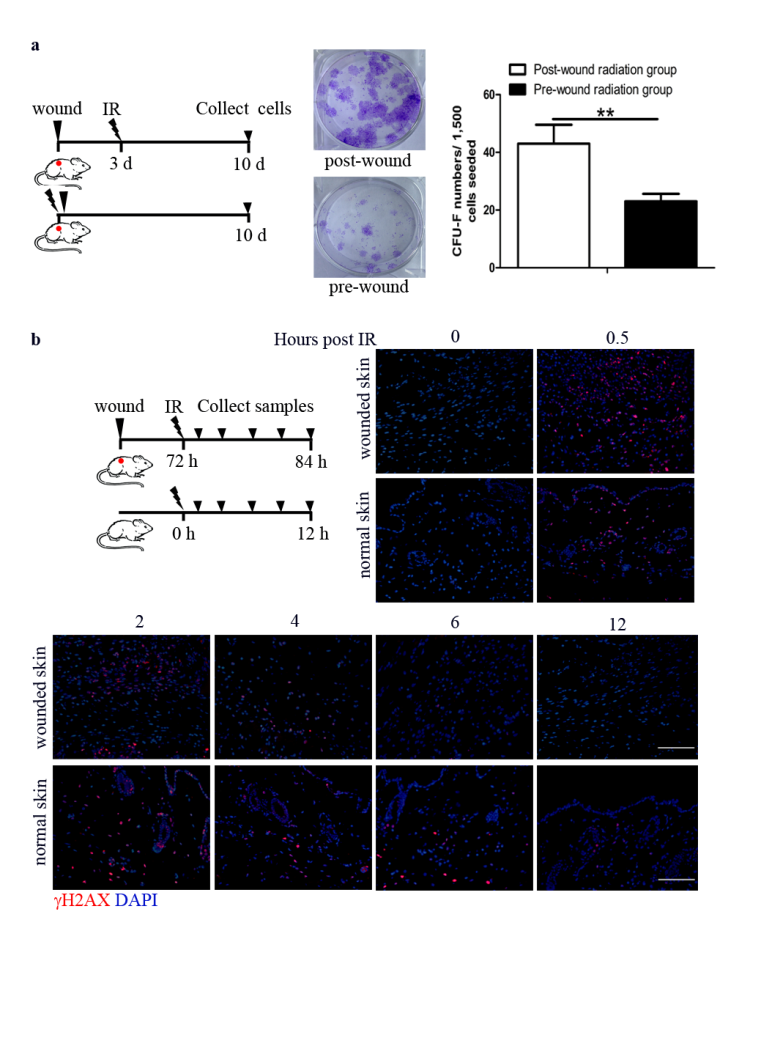
**

**Fig. S1. Mechanical injury enhances radio-resistance of skin fibroblasts in vivo.** (a) Mice in pre-radiation wound group were wounded 3 days before 6 Gy radiation, mice in post-radiation wound group were wounded immediately after radiation. Granulation tissue derived cells were harvested 10 days post wound. Clonogenic survival assay of granulation tissue cells were performed and colonies were quantificated. **, P < 0.01. P values were calculated using the independent-samples t test. (b) Full-thickness wound were made 3 days before radiation, wounded mice and normal mice then received 6 Gy radiation, the wound and normal skin tissue at indicated time points following radiation were harvested and stained for γH2AX. Cell nuclei were counterstained with DAPI. Scales bars are 200 µm.


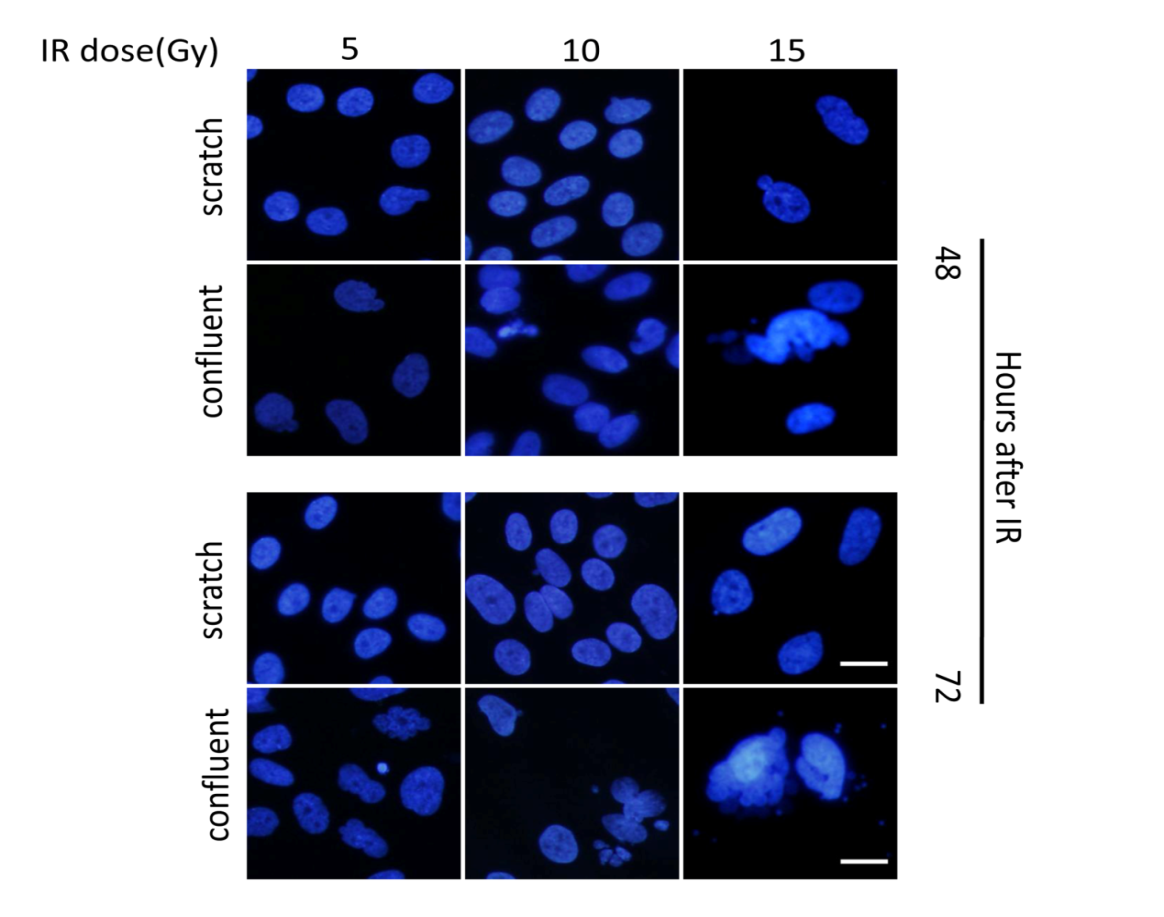


**Fig. S2. Mechanical injury decreases radiation induced micronuclei.** Representative pictures of micronuclei of mechanically scratched and confluent skin fibroblasts 48 and 72 hours following 5, 10 and 15 Gy radiation. Scale bars were 20 µm.


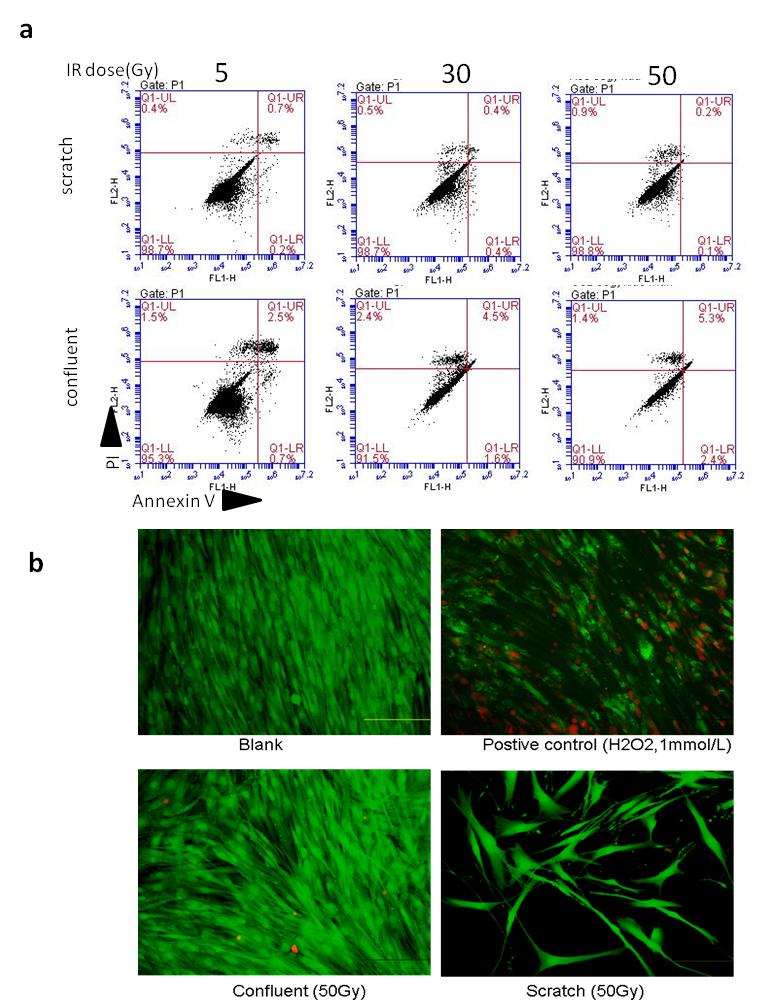


**Fig. S3. Mechanical injury reduces radiation induced apoptosis.** (**a**) Flow cytometric analysis of mechanically scratched and confluent skin fibroblasts 48 hours following 5, 30 and 50 Gy radiation. (**b**) Calcein-AM (green) /PI (red) staining of mechanically scratched and confluent skin fibroblasts 24 hours following 50 Gy irradiation. Scale bars: 200 µm.


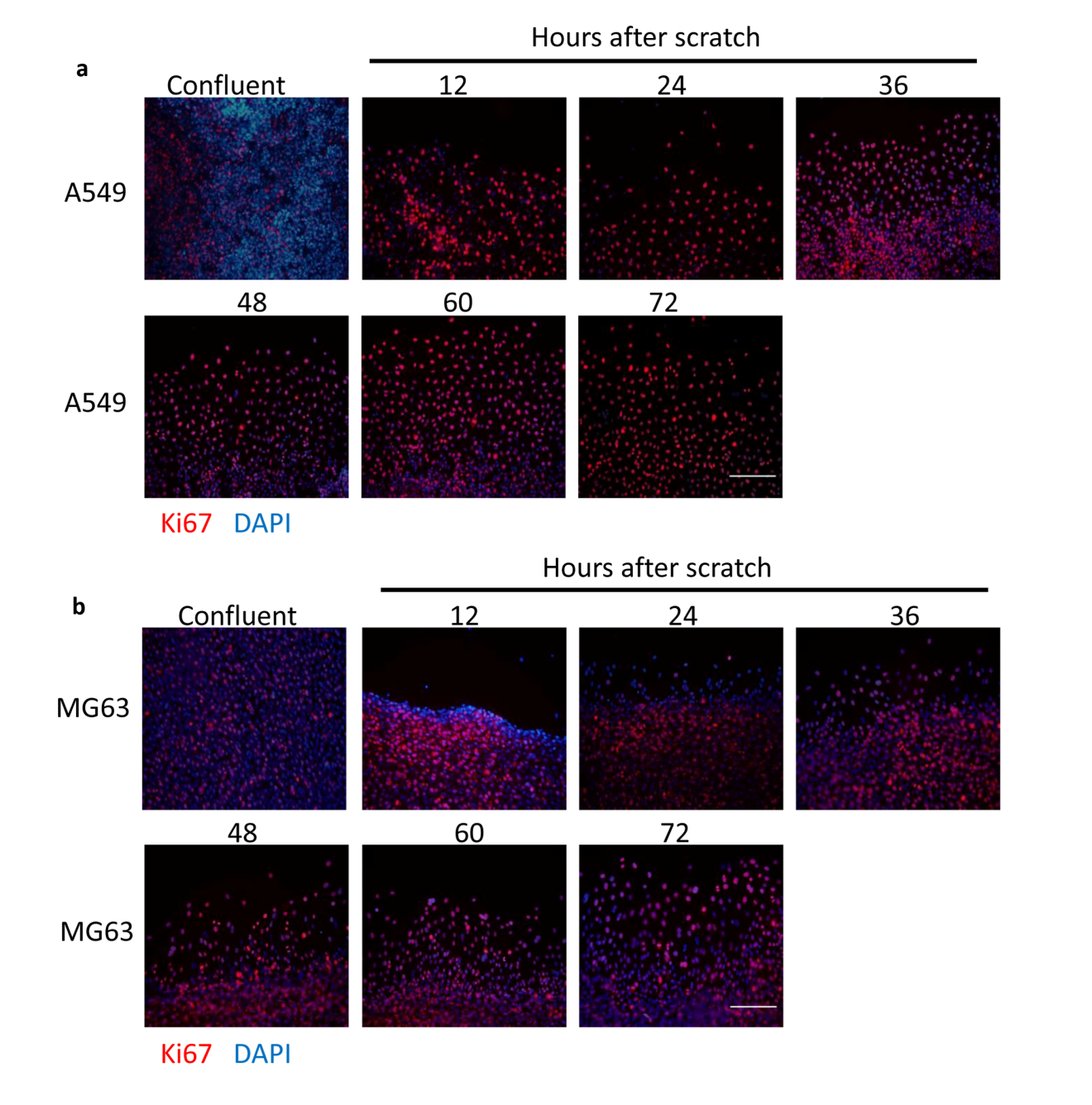


**Fig. S4. Patterns of Ki67 expression of tumor cells following mechanical injury are present.** (**a**) After achieving confluence over 7 days, A549 cells were mechanically scratched. Cells were then fixed at indicated time points and stained for Ki67. (**b**) Same as in a, but cells were MG63 cells. Scale bars were 200 µm.


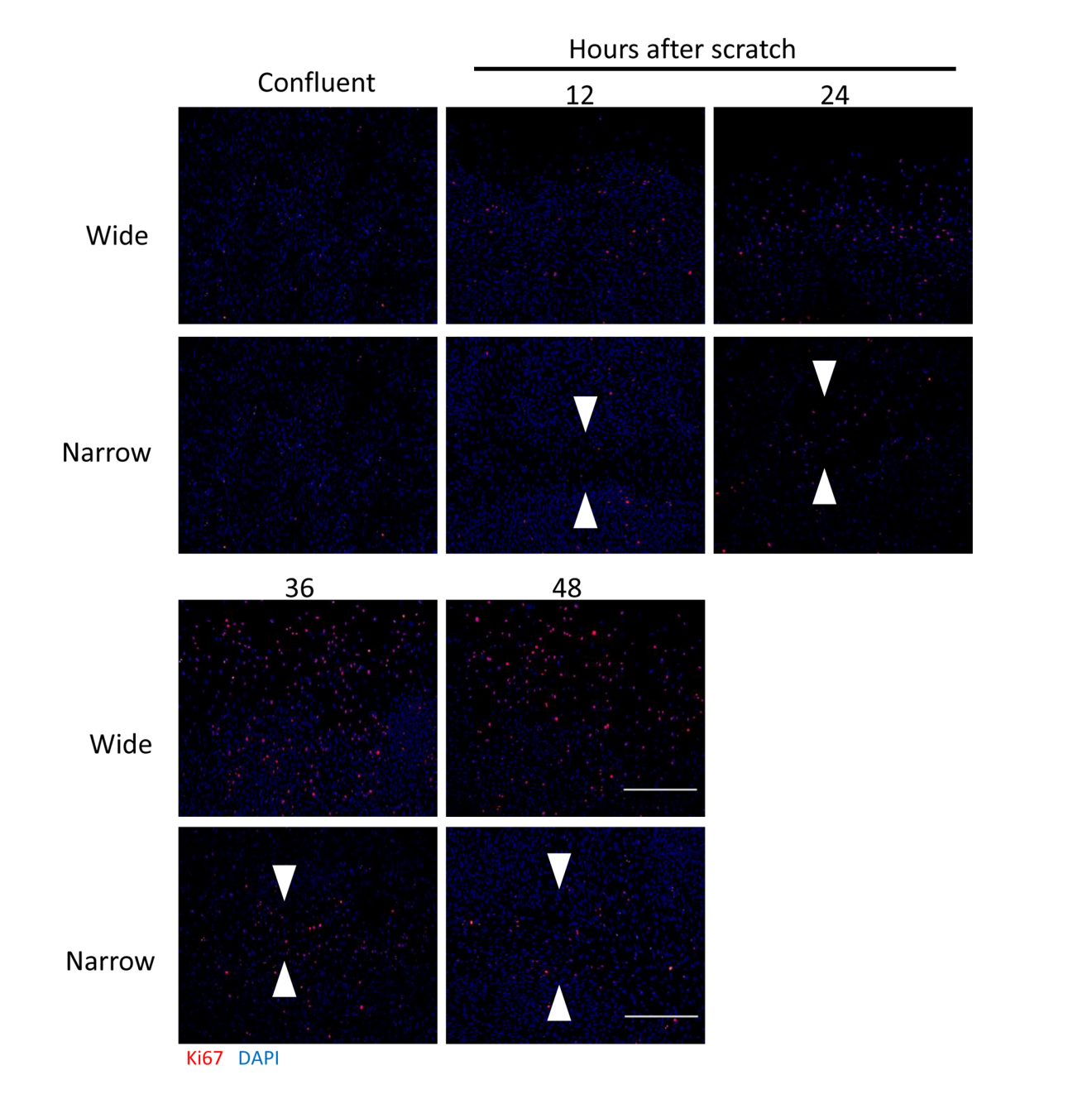


**Fig. S5. Patterns of Ki67 expression following different size of mechanical injury in vitro are present.** After achieving confluence over 7 days, skin fibroblasts were scratched with a 200 ml pipette tip (wide) and a 1 ml syringe needle (narrow) to mimic different wound size. Cells were then fixed at indicated time points and stained for Ki67. Nuclei were counterstained with DAPI. Scale bars were 500 µm. Arrowhead indicated scratch edges.

**
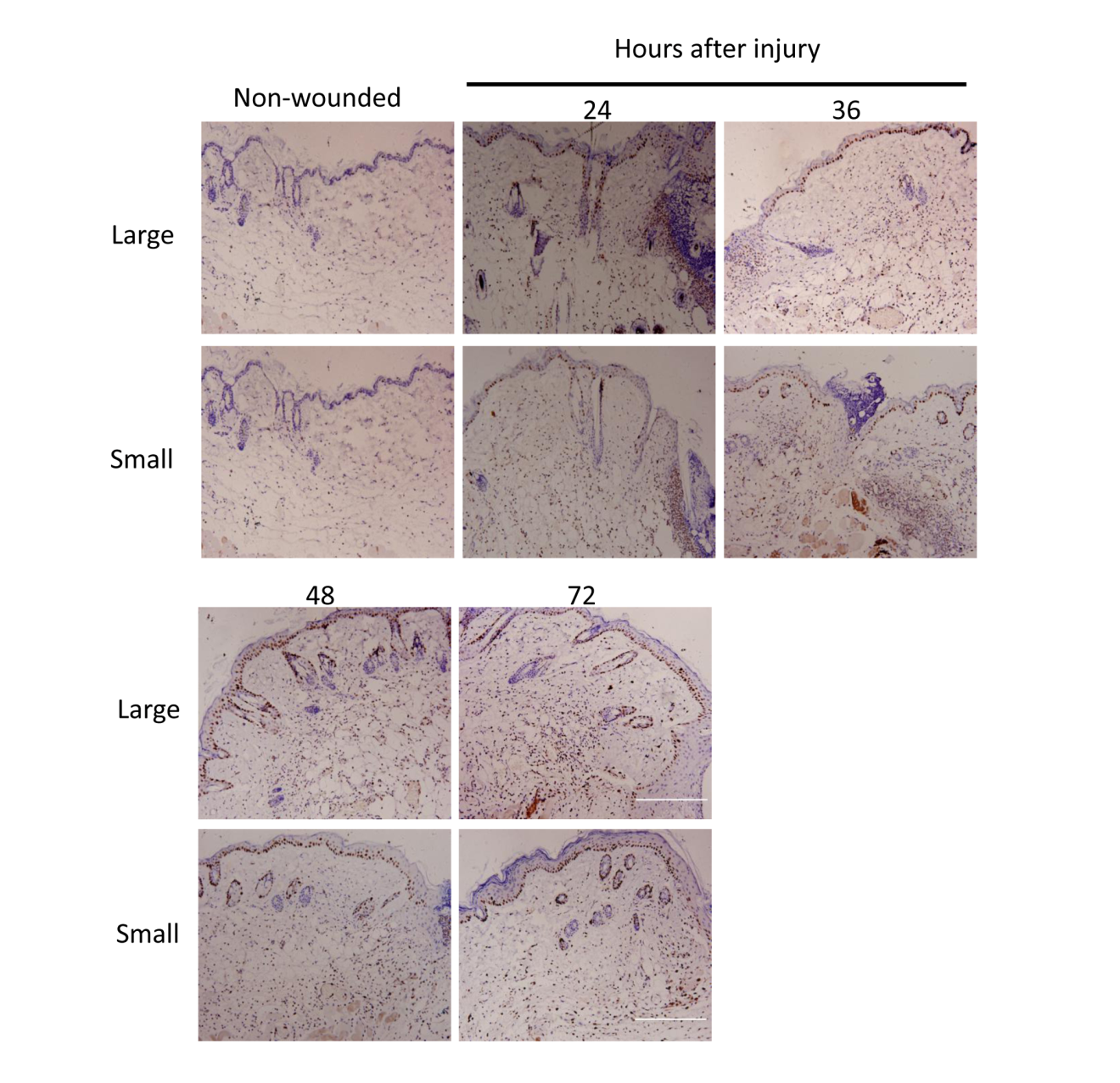
**

**Fig. S6. Patterns of Ki67 expression following different size of mechanical injuries in vivo are present.** Mice received a 1 cm in diameter full-thickness wound (large) and a line scratch wound with a scalpel (small). Wound tissues were harvested and fixed at indicated time points and immunohistochemistry stained for Ki67. Nuclei were counterstained with hematoxylin. Scale bars were 500 µm.


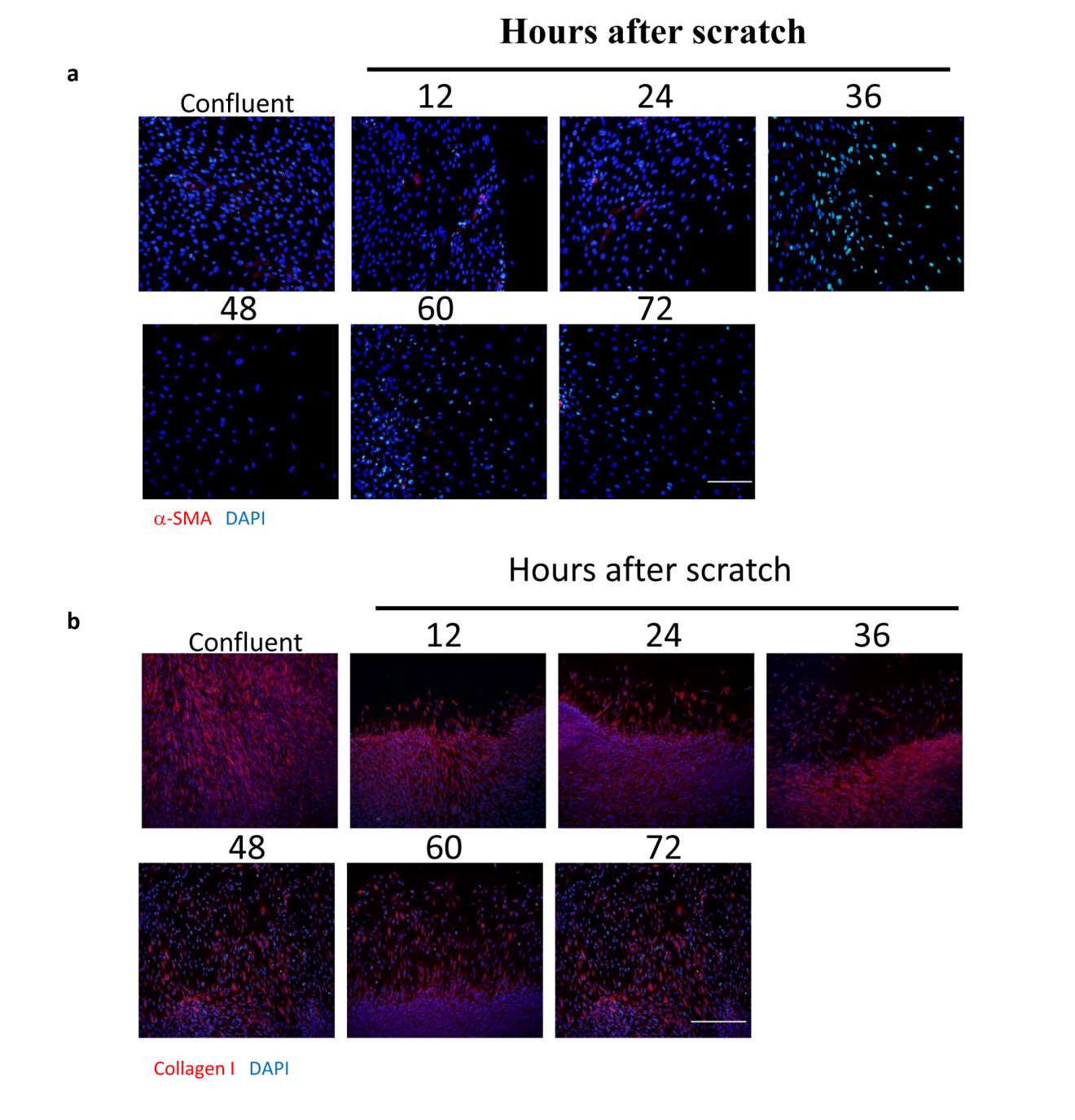


**Fig. S7. Patterns of a-SMA and Collagen I expression following mechanical injury are present.** (a,b) After achieving confluence over 7 days, skin fibroblasts were scratched. Cells were then fixed at indicated time points and stained for a-SMA and Collagen I. Scale bars were 200 µm.


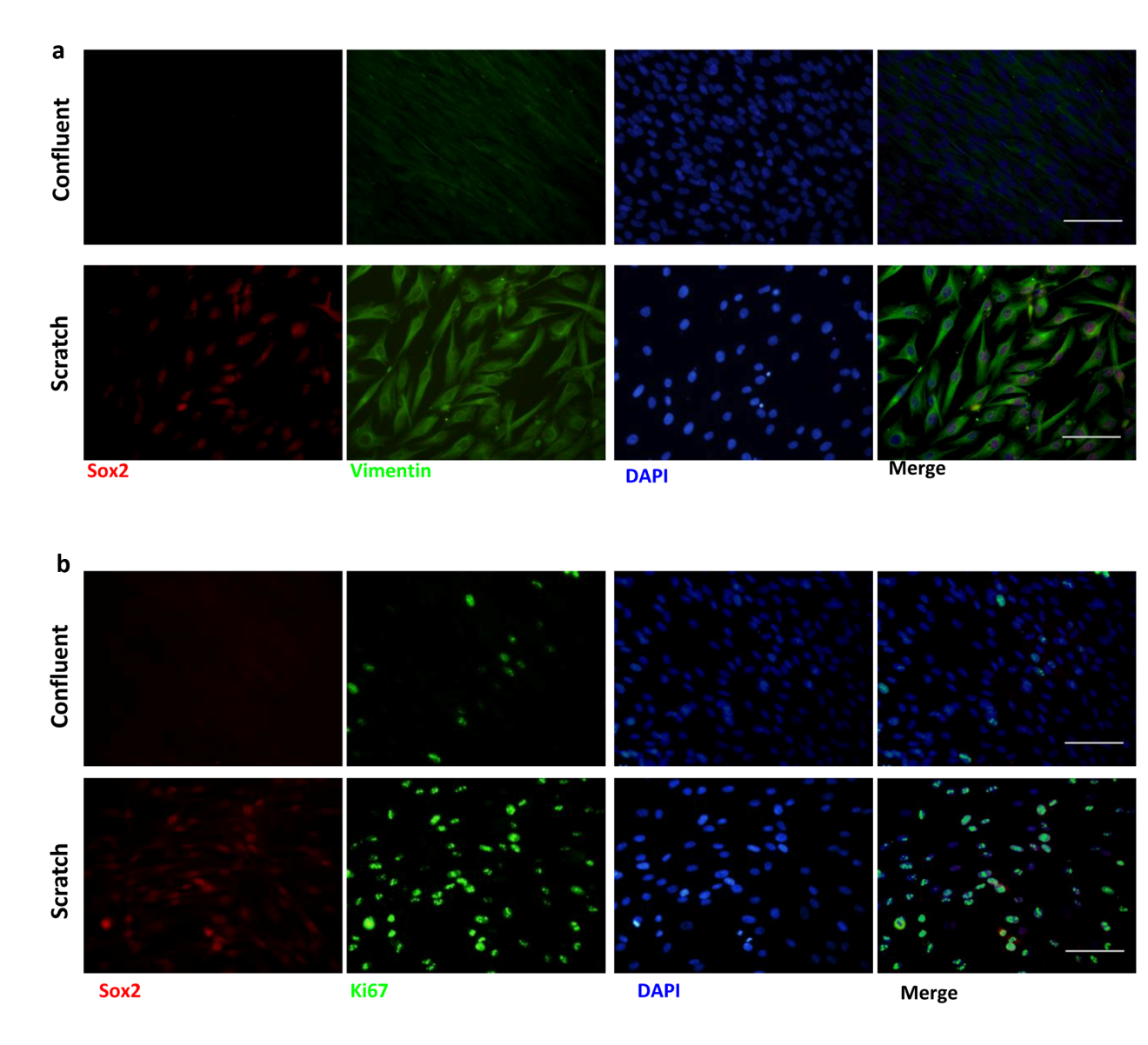


**Fig. S8. Phenotype of skin fibroblasts following mechanical injury is present.** (a) After achieving confluence over 7 days, skin fibroblasts were scratched. Cells were then fixed 72 hours following scratch and co-stained for Sox2 (red) and Vimentin (green). Nuclei were counterstained wit h DAPI. Scale bars were 100 mm. (b) Same as in A, but cells were co-stained for Sox2 (red) and Ki67 (green). Scale bars were 100 µm.


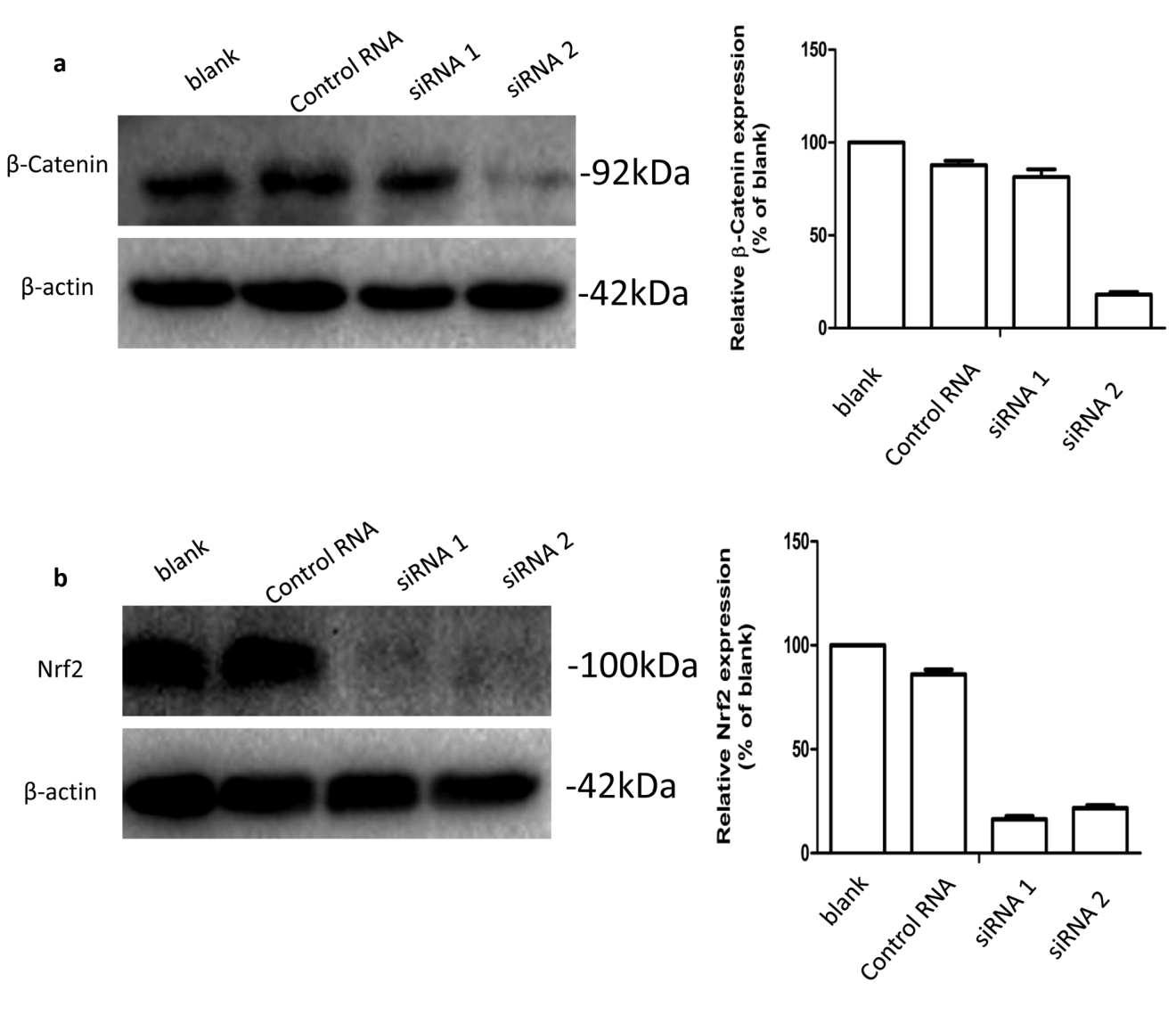


**Fig. S9. Knockdown of β-Catenin and Nrf2 following siRNA transfection are present.** (a) Western blot analysis and quantification of expression of β-Catenin following knockdown with siRNA constructs. (b) Same as in A, but expression of Nrf2 was tested.


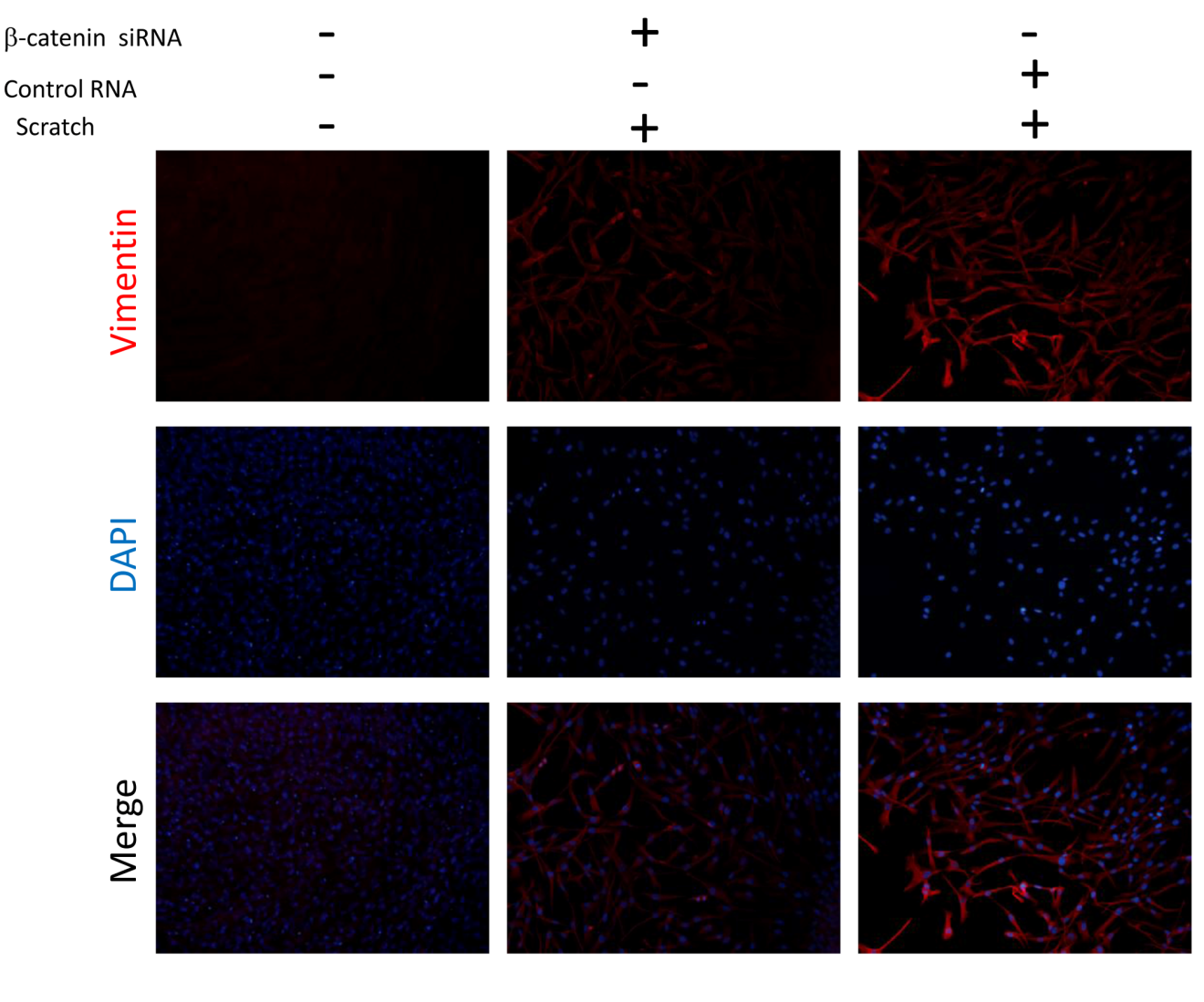


**Fig. S10. Patterns of Vimentin expression following knockdown of b-Catenin are present.** Immunoﬂuorescence staining for Vimentin of mechanically scratched and confluent skin fibroblasts following knockdown of b-Catenin. Scale bars were 200 µm


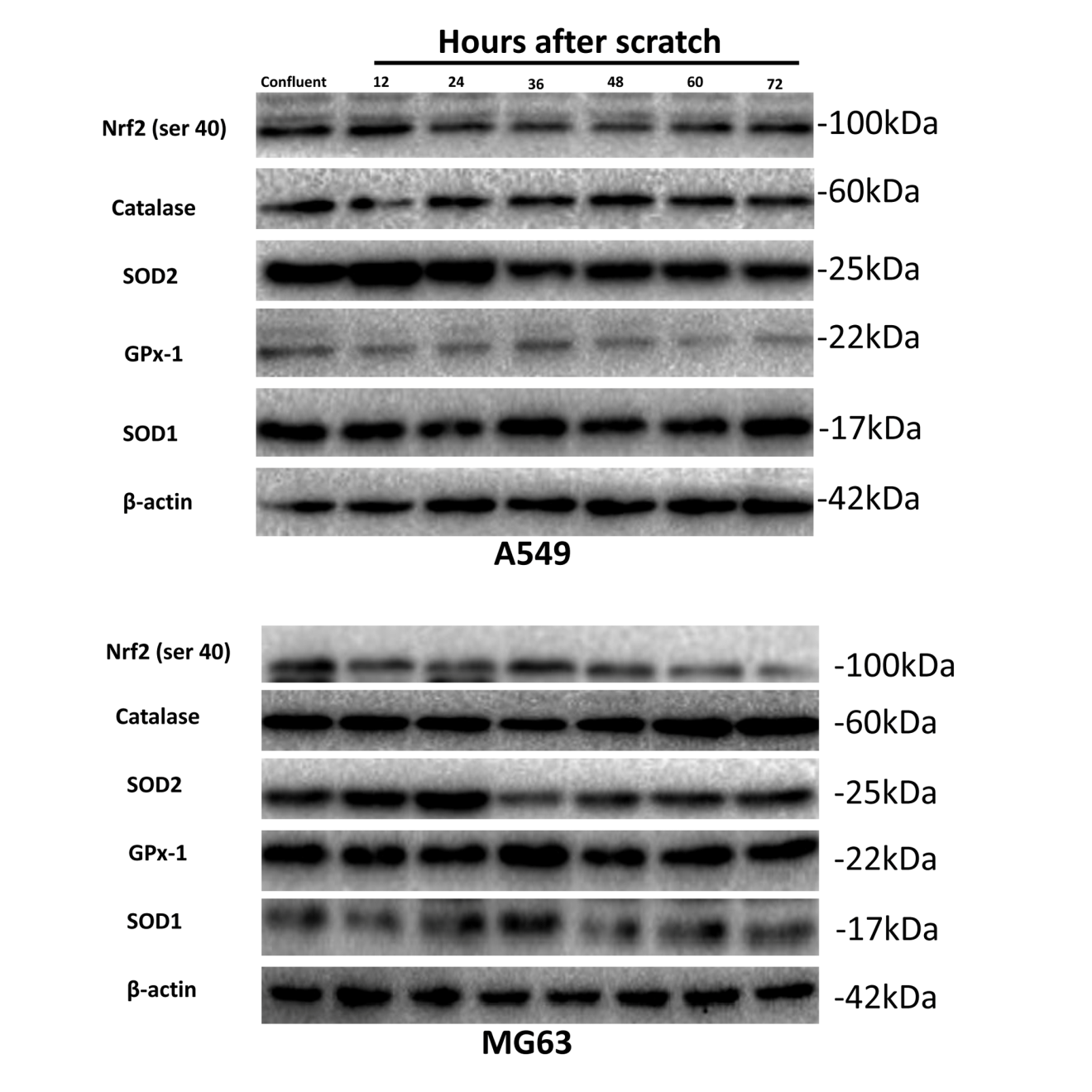


**Fig. S11. Patterns of antioxidant proteins expression following mechanical injury in tumor cells are present.** Western blot analysis of antioxdant proteins expression in A549 and MG63 cells at indicated time points following mechanical scratch.

**Supplementary tables**

|  | Scratch (normal) | | | Scratch (5Gy) | | |
| --- | --- | --- | --- | --- | --- | --- |
| Time (h) | G_1_（%） | S（%） | G_2_/M（%） | G_1_（%） | S（%） | G_2_/M（%） |
| 4 | 48.76±1.23 | 35.81±1.86 | 13.46±5.77 | 43.98±5.38 | 29.12±5.14 | 25.48±4.54 |
| 8 | 66.34±2.58 | 24.67±5.11 | 8.73±3.12 | 42.39±3.33 | 19.15±6.54 | 37.28±3.21 |
| 12 | 65.43±3.14 | 26.76±3.21 | 6.3±2.33 | 48.92±3.41 | 9.01±3.57 | 42.2±4.01 |
| 24 | 34.62±1.78 | 26.09±4.84 | 37.43±4.44 | 61.63±5.98 | 13.42±4.04 | 24.72±5.14 |
| 36 | 64.05±3.85 | 28.36±4.53 | 7.81±5.41 | 61.49±4.85 | 19.23±5.89 | 16.88±4.85 |
| 48 | 71.45±4.89 | 22.05±5.75 | 7.26±3.56 | 85.28±5.95 | 7.94±3.45 | 7.07±1.34 |
| 60 | 72.82±2.46 | 22.45±3.77 | 4.93±2.67 | 81.53±4.66 | 10.11±4.78 | 9.28±2.48 |
| 72 | 82.37±5.43 | 12.51±7.75 | 4.73±2.66 | 67.65±3.84 | 23.73±6.22 | 7.61±2.35 |
|  | Confluent (normal) | | | Confluent (5Gy) | | |
| Time (h) | G_1_（%） | S（%） | G_2_/M（%） | G_1_（%） | S（%） | G_2_/M（%） |
| 4 | 87.84±4.05 | 7.49±1.89 | 4.04±1.24 | 82.44±4.98 | 13.33±3.75 | 5.2±2.44 |
| 8 | 83.01±5.11 | 13.87±2.42 | 3.77±2.77 | 81.31±5.74 | 11.96±2.65 | 5.53±1.77 |
| 12 | 80.27±5.41 | 16.01±2.46 | 4.15±1.23 | 80.1±6.42 | 12.7±2.02 | 3.06±1.25 |
| 24 | 41.66±6.56 | 43.03±2.72 | 13.94±1.78 | 83.12±5.89 | 13.32±3.14 | 3.68±1.41 |
| 36 | 44.08±4.74 | 24.5±5.44 | 30.95±3.24 | 78.94±4.47 | 7.41±3.02 | 10.38±2.68 |
| 48 | 62.06±5.98 | 25.57±3.32 | 10.49±2.69 | 84.44±3.13 | 8.09±3.85 | 7.34±2.87 |
| 60 | 77.38±5.13 | 15.16±4.85 | 8.35±2.47 | 87.64±5.01 | 6.5±3.68 | 7.26±3.78 |
| 72 | 83.03±2.88 | 12.06±3.98 | 6.07±2.11 | 88.99±4.87 | 6.10±1.95 | 6.261.38 |

**Table S1. Cell cycle analysis of human dermal cells post IR.**
